# Reducing private car demand, fact or fiction? A study mapping changes in accessibility to grocery stores in Norway

**DOI:** 10.1186/s12544-021-00500-7

**Published:** 2021-07-05

**Authors:** Lillian Sve Rokseth, Eva Heinen, Espen Aukrust Hauglin, Tobias Nordström, Bendik Manum

**Affiliations:** 1 Department of Architecture and Technology, Norwegian University of Science and Technology, Trondheim, Norway; 2 The Institute for Transport Studies, University of Leeds, Leeds, England; 3 Institute of Urbanism and Landscape, the Oslo School of Architecture and Design (AHO), Oslo, Norway; 4 Spacescape AB, Stockholm, Sweden

**Keywords:** Accessibility, Car ownership, Grocery shopping, Geographic information systems, Active mobility

## Abstract

**Background:**

Travel surveys show that the amount of private car driving in Norway has increased significantly since the mid-1980s. Private car driving has for a long time been the main mode of transport for retail and service trips, and grocery shopping trips represent over 60% of the retail and service travels. Despite the growing number of studies addressing accessibility to daily destinations, to the best of the authors’ knowledge there are no studies examining these issues over time.

**Methods:**

This paper aims to investigate changes in accessibility to grocery stores over time and use two counties in Norway as examples. Based on GIS data at a detailed level, distances from dwellings to nearest grocery store has been examined.

**Findings:**

The results from the spatial analyses reveal significant changes from 1980 to 2019: The share of the population living within 500-m from a grocery store has decreased from 55% to 34% in one of the counties examined and from 36% to 19% in the other. This indicates that the share of people living within walking distance to a local grocery store has nearly halved. With such changes in accessibility to grocery stores, increased car driving for grocery shopping should not come as a surprise. Contrary to the frequent statements about sustainable urban development and active transportation, it seems that Norway still is developing as a country that in the future will be more and not less dependent on private cars.

## Introduction

There is growing consensus that we need to reduce car use and increase the share of walking and bicycling based on a variety of reasons, ranging from improving public health, creating attractive cities, and reducing greenhouse gas emissions, pollution and noise. This aim is formally stated by key international institutions like UN-IPCC, UN-Habitat, and WHO, by governments, as well as by numerous actors at city scale across the world [28, 37, 38]. In Norway, therefore, any future increase in personal travel is aimed to take place in public transport, walking and cycling instead of car use [24, 25]. In order to achieve this there is much emphasis on the planning, designing, and building of cities and neighbourhoods to ensure that peoples’ daily lives can become less dependent on cars.

Despite these intentions regarding sustainable urban development and reducing car driving during the recent decades, most households in Norway own a private car (87% of Norwegian households in 2018 [36];) and car driving is the dominant mode of travel, representing 55, 52, and 53% in national travel data surveys from 2013/2014, 2018 and 2019 respectively. Regarding the different purposes of travel, retail and service travels represented the largest share (28%) of all travel types for Norwegian households at National level in 2018, even after commuting (21%) [36]. Due to the low population density in Norway, the main mode of transport for retail and service trips has for a long time been private car driving [9], and grocery shopping represent over 60% of the retail and service travels [13]. Over time the national travel data surveys show that car use has increased significantly since the mid-1980s. Between 1985 and 2013 (the last detailed national travel data survey published) the average total distance driven per person for shopping travels has increased as much as 76% [13].

To reduce car use, it is necessary to improve the conditions for people to manage their daily lives without the private car. People’s travel mode choices are closely intertwined with their choice of place of work, choice of place to live as well as the locations of their other important frequent destinations. For some, workplace together with options of travel determine decisions about where to live, whereas for others, a beloved place of living may determine options of work as well as mode of travel. In general, as pointed out by Marchetti [22], peoples’ choice of travel mode is a result of priorities within households’ budgets of time and economy, leading to “Marchetti’s constant” of 1 hour of travel a day being the case through human history. Regarding modes and distances, the travel distance between origin and destination impact on the probability to choose a particular mode of travel [3, 5, 8]. Different distances favour some travel modes and make other modes irrelevant; on the extremes, intercontinental business travels are difficult without airplanes, whereas walking is hard to beat for any purpose for the shortest trips [13]. Even though options of web ordered at home delivery of food has increased during the Covid-19 pandemic, the key transport mode options for buying food in Norway are private car or walking, and the distance to grocery stores will be impacting on mode choice for shopping directly.

One reason for increased dominance of the car for shopping trips may be changes in function mixing in cities, specifically the location of shopping. Cervero [4] found that vehicular trips for shopping in US could decrease by 25% if retail and service were located close to residences. Jiao, Moudon, and Drewnowski [16] identified longer distances between homes and grocery stores as one of the strongest predictors for travellers choosing car driving as travel mode for grocery shopping. This is supported by Schneider [27] in a study of 20 shopping districts in the San Francisco Bay Area. In a study focussing on healthy food accessibility, Li and Kim [18] found higher accessibility to be associated with shorter distances from dwellings to grocery destinations. Other studies, however, have found that residents in areas with higher accessibility to shopping destinations likely will have a higher travel frequency [10] and make more one-stop shopping trips than those with poorer access [1, 19].

To understand the development towards more car driving, a key issue is how origin- and destination issues important for car demand have changed over time. Despite popularity of concepts like “city of short distances”, the 10-min city and mixed used neighbourhoods (See for example [7, 14, 15]), to the best of the authors’ knowledge there are no studies examining these issues over time.

The aim of this paper is to examine how residential locations and locations of grocery stores relative to one another have developed in Norway over time and the consequent implication of accessibility to grocery stores. For this, we investigated the situation in 1980 and 2019 in two counties in Norway: Hommelvik and Lørenskog by means of geographical information systems (GIS) data and spatial analysis. Accessibility is in this paper used to describe *“the spatial distribution of potential destinations”* and *“the ease of reaching each destination”* ([11], p. 1175) and is measured as network distance between dwelling and grocery store. Regarding causalities between accessibility and mode choice, residential proximity to grocery stores does not imply that people walk (or bicycle) for daily shopping (as people may still go by car) [12], whereas the opposite is in general the case; long distance to grocery store implies going by car. Therefore, the focus in this paper has been to examine the shares of the population not having grocery stores within distances convenient for walking or bicycling. If travel distances for shopping show to have increased over time, this can be part of the explanation why car use has not decreased as expected by politicians and other stakeholders and can also provide knowledge about how to change our built environment in order to contribute to less car driving and more active mobility.

This paper consists of five parts. In the following section the method applied in the study is described. Section 3 presents results from the GIS analyses. Section 4 discusses results, while section 5 offers conclusions and implications for policy derived from the analyses.

## Method

### Study area

**Fig. 1:**
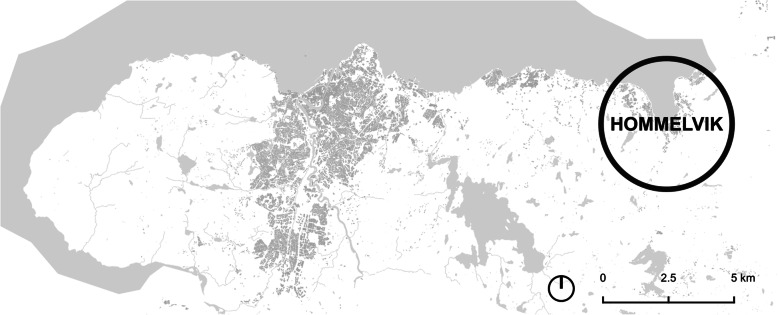
The Trondheim region, with Hommelvik

**Fig. 2:**
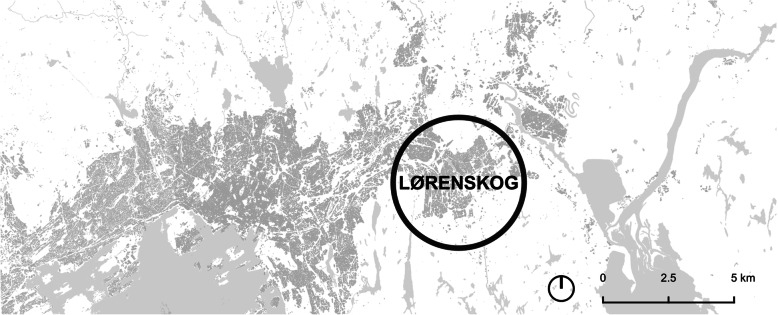
The Oslo region, with Lørenskog

This study was conducted during the fall semester in 2019 as part of a GIS course for architecture students where the task of the students was to examine development of accessibility to grocery stores in their hometowns. In this paper, we present the studies of Hommelvik, west of Trondheim (Fig. 1) and Lørenskog, neighbouring Oslo (Fig. 2).

Hommelvik, 24 km west of Trondheim, is the administrative centre of Malvik municipality. Malvik has about 14,000 inhabitants, whereas Hommelvik, covering an area of 2,9 km^2^, has a population of 5501 inhabitants [33]. Hommelvik was established around sawmill industry, but the majority of the local workplaces today are within administration and service activities. During and after the 2nd World War, an extensive share of holiday houses for inhabitants of Trondheim were built in the Malvik area. Later, a suburban housing development has taken place because of the close connection to Trondheim, as a large share of the inhabitant’s commute to work in Trondheim (62% in 2016) (Store Norske [17]).

Lørenskog is located 14 km east of Oslo, stretches over 71 km^2^ and has a population of 42,106 inhabitants [33]. Lørenskog has developed around sawmill industry in the south, agriculture in the middle, while the areas along the railroad and the main roads in the north were dominated by suburban housing. The suburban area spread further south as the population increased. A large share of the population commuted to work in Oslo, but with the establishment of a new hospital in 1960 and a shift to trade and service industries, the share of commuters decreased [20]. In 2020, 36% of the population commute to work outside the municipal area [32].

### Data

The analyses are conducted in GIS and are based on a GIS model containing the walking- and bicycling networks, the grocery stores as well as the population located by dwellings. For the present situation, shapefiles with buildings and roads were retrieved from the Norwegian online map catalogue [23]. Due to confidentiality constraints some adaptions were required for providing data at the level of detail needed for performing the analyses. This section describes the data collected for the grocery stores, for the population, and for the walking- and bicycling networks, respectively.

#### Grocery stores

For the current situation, i.e. 2019, the grocery stores were mapped based on municipal maps, open street map data [26] and local knowledge. The stores were positioned in GIS as a point in the centre of the building (i.e. floor plan) in which the store is located. For the 1980 dataset, the mapping of stores consisted of three steps. Starting with the 2019 map, the students together with their parents and others with local knowledge used older maps to trace the condition back in 1980. First, buildings built after 1980 and hence also the stores located within these building were deleted. Second, stores that existed in 1980 and had closed down since were added. Finally, local authorities and organisations such as local history societies were consulted for confirmation and completion of the information.

#### Population data

Existing data at local and national level provided by the Norwegian mapping authorities are very detailed. They map every person, and associated characteristics to a particular dwelling’s geolocation. Due to confidentiality constraints, this information cannot be accessed without special permits. Publicly available population data is available as gridded data at a resolution of 250 × 250 m [35]. Instead of aggregating other data to the same grid, we “disaggregated” the aggregated data on residents per grid cell into persons per residential building. This was done by distributing the population of each grid cell (the 250 × 250 meter units) equally over the residential buildings within the same grid, where residential buildings are found by codes given in the Norwegian real estate register, the so called GAB registry [34]. As a result, the residential dwellings within each grid cell house the same number of people, regardless of the real number of dwellings in a building, type of building etc., variables that not only vary a lot, but that are also interconnected [21]. However, despite some simplification, our modelling positions the population in dwellings at the real address points of the dwellings, and is therefore a much more realistic GIS mapping of residents than threating the population as one number located at the centre of each grid cell or distributing them evenly over the entire area of each grid cell, regardless of the area being house, road, forest or sea.

The population data layer of 1980 was made by deleting all residential buildings built after 1980 from the 2019 GIS model and thus excluding also the residents in those buildings. Since 1980, the average floor area (dwelling area) per person has, according to the EU-SILC (Statistics on Income and Living Conditions) survey, increased from 36 m2 to 58 m2 in 2013 [30] and the size of households (by number of persons) has decreased from 2.66 to 2.16 in 2019 [31]. This means that a neighbourhood over a time period will have significant change in population density, even if dwellings, grocery stores and street networks remained constant. If mapping changes for individual people over time from social sciences or public health points of view, the above-mentioned differences in living conditions over time should be included. For comparing how built environment perform regarding accessibility at different times, the significant influence of changing living standard and household compositions would represent a bias. Therefore, since the aim of this paper is the latter rather than the former, we chose to measure the accessibility of the built environment of 1980 populated in accordance with today’s living standard. By doing so, we did not analyse development of the number of people and their accessibilities. Instead, we have provided a measure (a population count) that works as a proxy for accessibility from dwelling units.

#### The route network

The route network in 2019 in GIS was made on the basis of the road centreline map (Vbase) provided by the Norwegian Mapping Authority [23], and Open Street Map [26]. The route network, along which distances are measured, are the routes for walking (and bicycling), including short cuts such as paths across a park, and excluding roads not allowed to walk on. Details in the routes were controlled based on personal knowledge of the place and with orthophotos (digital photo) provided by the local authorities. We added missing short cuts, and corrected other inconsistencies, such as connecting parts of the network that had missing links. The 1980 route network was made by deleting areas that has been constructed after 1980, and then controlling more in detail with maps from 1980.

### Analyses

**Fig. 3:**
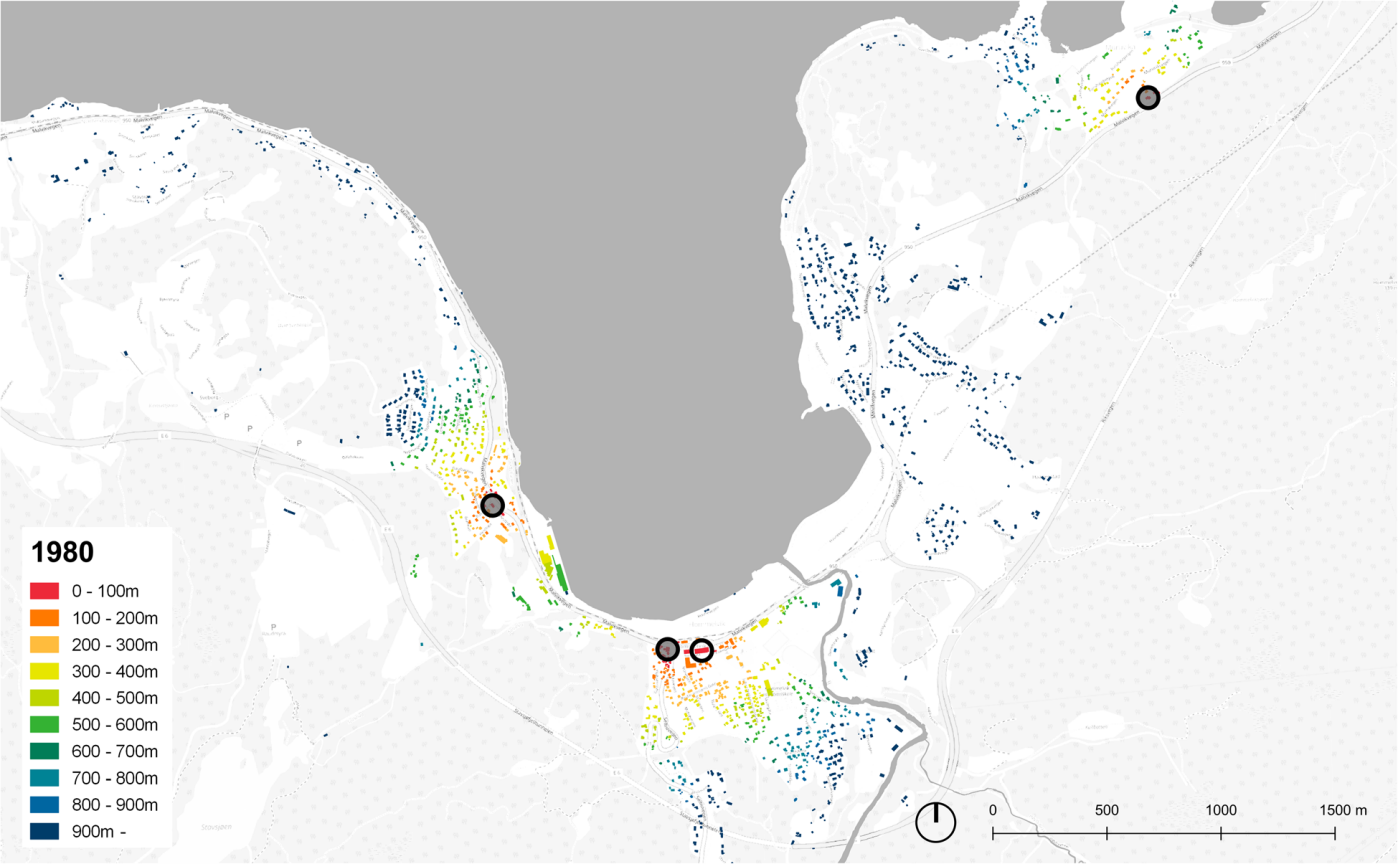
Distance to grocery stores, Hommelvik, 1980. Stores marked with circles, filled circles representing stores closed down since 1980

**Fig. 4:**
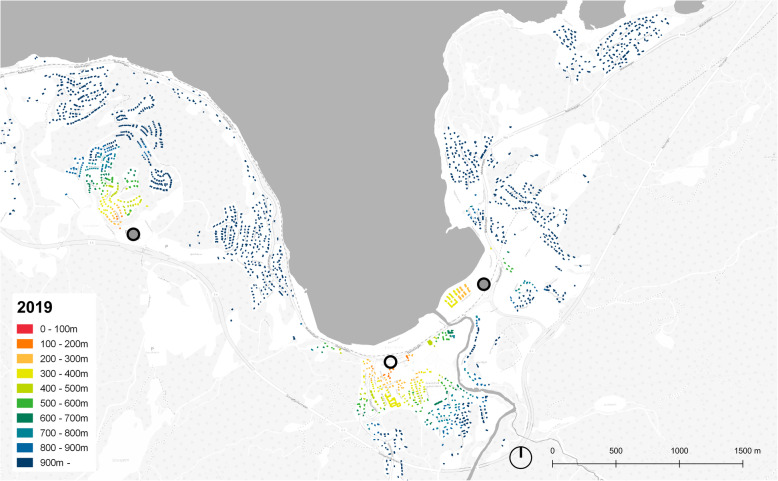
Distance to grocery stores, Hommelvik, 2019. Stores marked with circles, filled circles representing stores opened since 1980

**Fig. 5:**
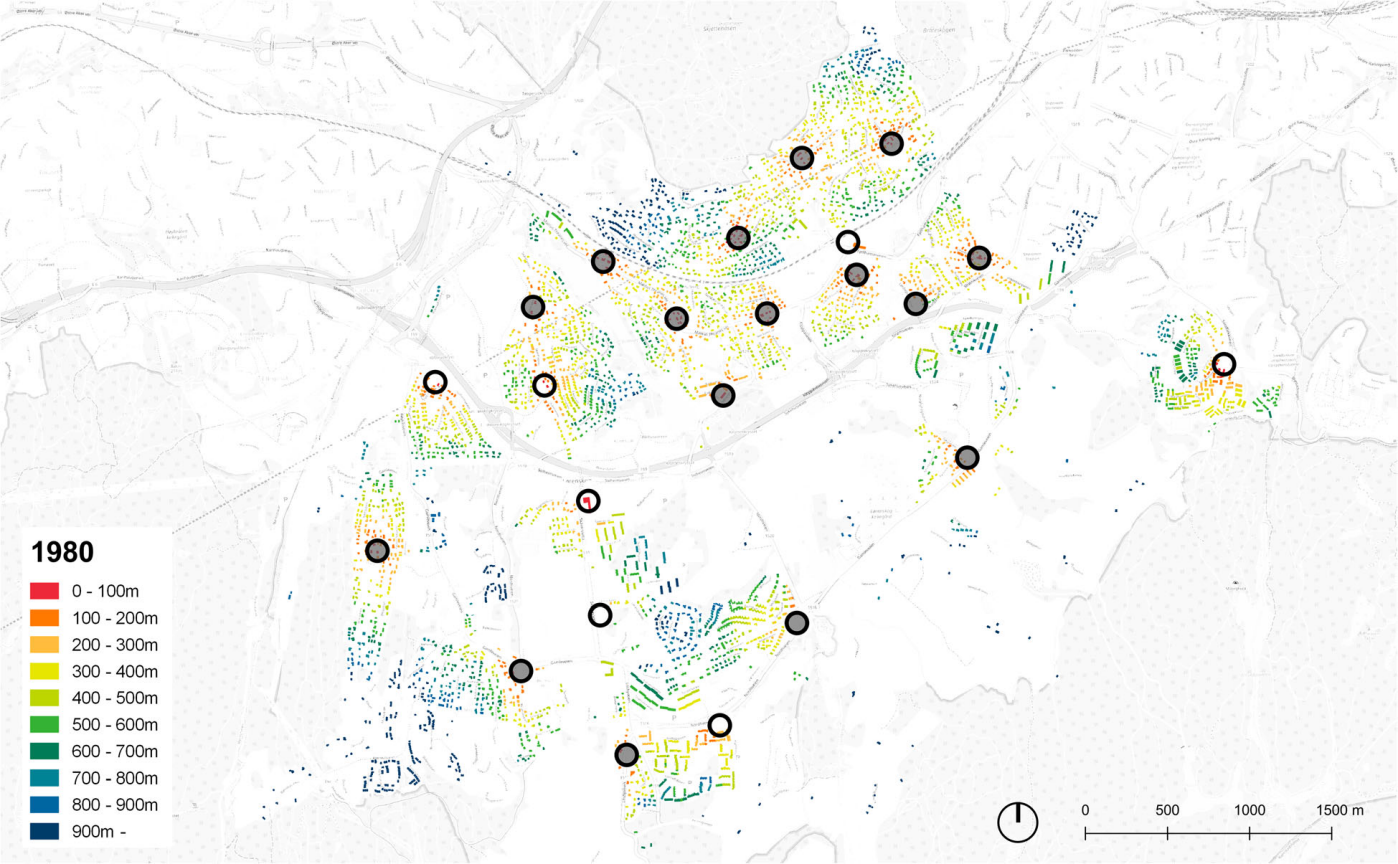
Distance to grocery stores. Lørenskog, 1980. Stores marked with circles, filled circles representing stores closed down since 1980

**Fig. 6:**
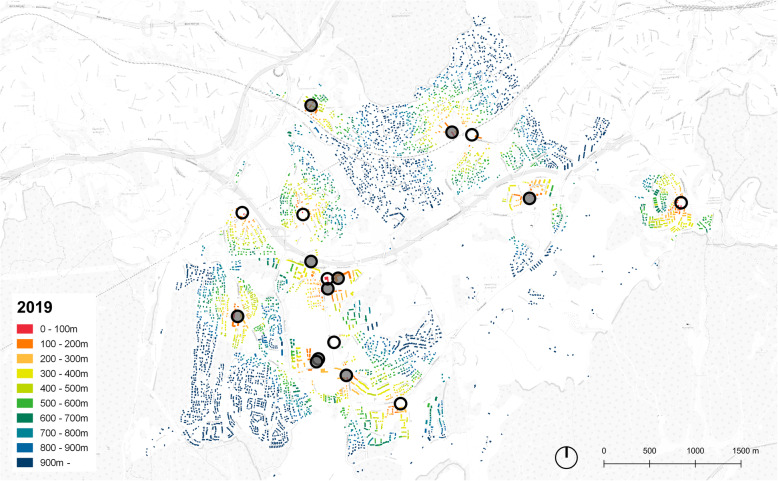
Distance to grocery stores, Lørenskog, 2019. Stores marked with circles, filled circles representing stores opened since 1980

The analyses were conducted using the GIS software QGIS and the plugin Place Syntax Tool [29], applying the function “attraction distance” where the distance between destination(s) and origin(s) can be extracted in various ways. We applied “attraction distance” to calculate the minimum walking distance (i.e. the network distance) in meters from each residential building (from the point at the centre of the residential building) to the closest grocery store (i.e. to the point at centre of a building with grocery store). The algorithm on which the “attraction distance” calculation is based, link each point to the closest line in the route network [29]. The calculations were conducted for the two situations, 2019 and 1980, separately, using the GIS layers created for each situation described previously as origins, networks and destinations. As an output of the “attraction distance” calculation, a new attribute column with values in meters to the closest grocery store was assigned to the GIS layers containing the residential buildings, which in turn were used to colour the maps presenting the situation in 1980 and 2019 for both Hommelvik and Lørenskog (Fig. 3, 4, 5, 6) and for extracting descriptive statistics as well as the number of people living within different threshold distances.

We used t-tests to compare the means between 1980 and 2019 for both cases. Chi-square tests were conducted to explore the relationships between the numbers of residents within distance groups in 1980 and 2019. Both the t-tests and the chi-square tests were conducted using IBM SPSS Statistics 27.

## Results

The basis of the results is the walking distance from each resident to the nearest grocery store. Table 1 show descriptive statistics comparing the situation in 1980 and 2019 for both Hommelvik and Lørenskog. The mean walking distance has increased from 1980 to 2019 in both cases, with 270 m in Hommelvik and close to 200 m in Lørenskog. As illustrated by the maximum values, as well as results in Table 2, a part of the population lives far from a grocery store, more than 3000 m in Hommelvik, and more than 5000 m in Lørenskog. Based on this, there is a potential for the mean to be skewed. However, the median values also show a considerable increase from the situation in 1980 to 2019, about 400 m higher in Hommelvik and less than 200 m higher in Lørenskog. Independent samples t-tests were carried out to examine whether there were differences in the mean walking distances between 1980 and 2019. The results were statistically significant in both Hommelvik: t(5665,289) = − 15,739, *p* < 0,001, and Lørenskog: t(54,664,085) = − 60,278, p < 0,001 indicating a significant increase in walking distance to grocery stores.

**Table 1:** Descriptive statistics of walking distance to nearest grocery store 1980 and 2019

	Hommelvik		Lørenskog	
	1980	2019	1980	2019
N	2846	6131	22,187	38,553
Mean	985	1257	540	736
Median	744	1139	473	654
Standard Deviation	757	775	351	439
Minimum	35	103	17	20
Maximum	3772	3484	5120	5657

**Table 2:** Numbers and percentage of population within different walking distances in 1980 and 2019

	Hommelvik				Lørenskog			
	1980		2019		1980		2019	
Distance threshold	n	%	n	%	n	%	n	%
< 500	1019	35.8%	1159	18.9%	12,174	54.9%	13,212	34.3%
500–1000	801	28.1%	1468	23.9%	8250	37.2%	16,175	42.0%
1000–1500	307	10.8%	1741	28.4%	1348	6.1%	7379	19.1%
1500–2000	339	11.9%	674	11.0%	261	1.2%	1422	3.7%
2000–2500	316	11.1%	488	8.0%	85	0.4%	236	0.6%
2500–3000	2	0.1%	432	7.0%	21	0.1%	48	0.1%
3000 <	62	2.2%	169	2.8%	48	0.2%	81	0.2%
SUM	2846	100%	6131	100%	22,187	100%	38,553	100%

Figure 3 shows the case of Hommelvik in 1980, where all residential buildings are coloured in accordance with walking distance to grocery store and where red means short distance. Comparing with the map of 2019 (Fig. 4), several aspects of the development during the time period stands out. One issue is local stores within short walking distances being closed down (see the area by the fjord, some north-west of the centre of Hommelvik, and the area at the upper north-east of the maps). A second issue is new single-family house areas being developed without being offered grocery stores (at the upper north-west of Hommelvik). In Lørenskog in 1980, the grocery stores are evenly distributed all over the city (Fig.5), very different from the current situation where grocery stores are located more in clusters (see the central area of Lørenskog, Fig. 6). This may lead to reduced prices of food due to competition, but not to increased accessibility to nearest store.

**Fig. 7:**
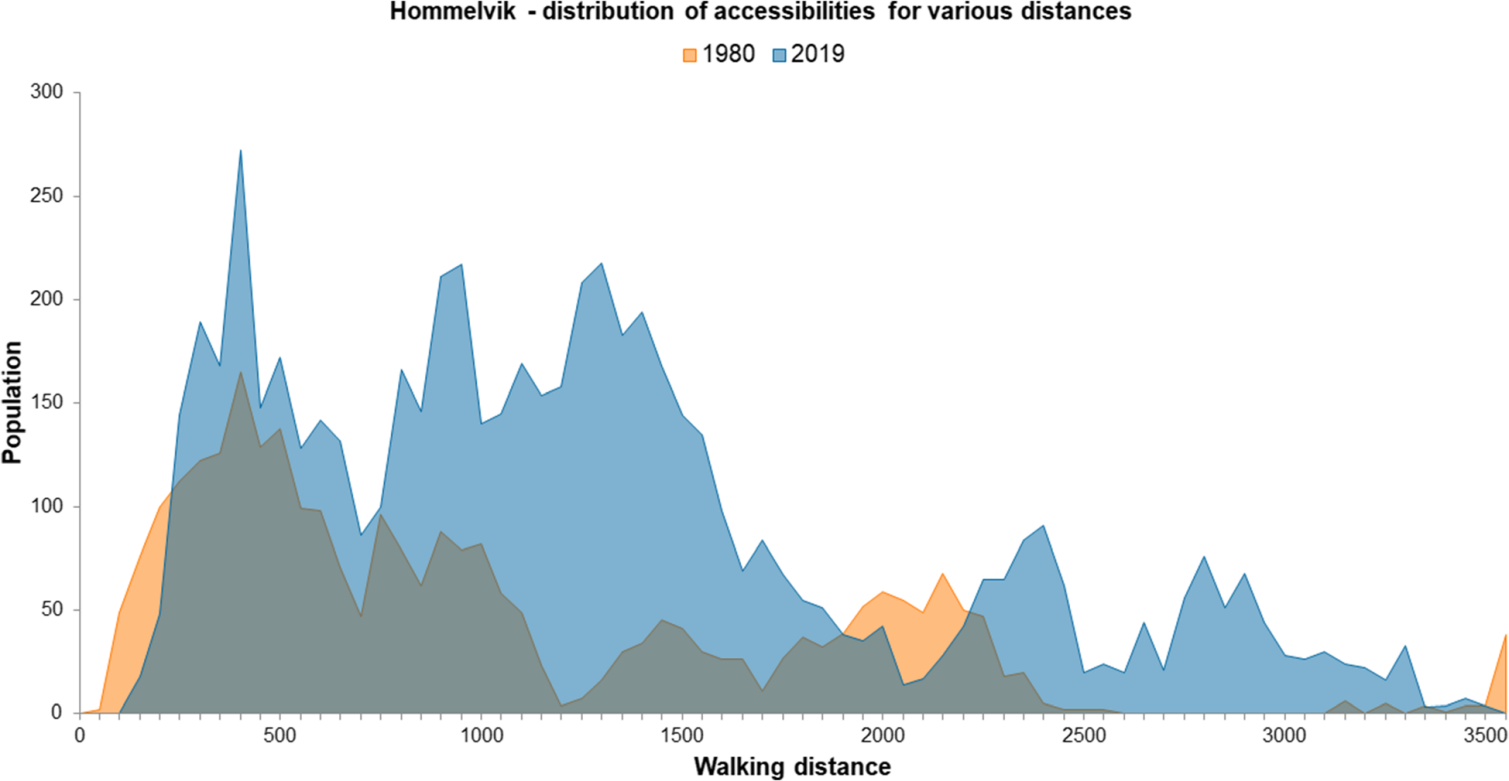
Accessibility from dwellings to grocery stores in Hommelvik. Population at different distances in 50 m intervals, 1980 (orange) and 2019 (blue)

**Fig. 8:**
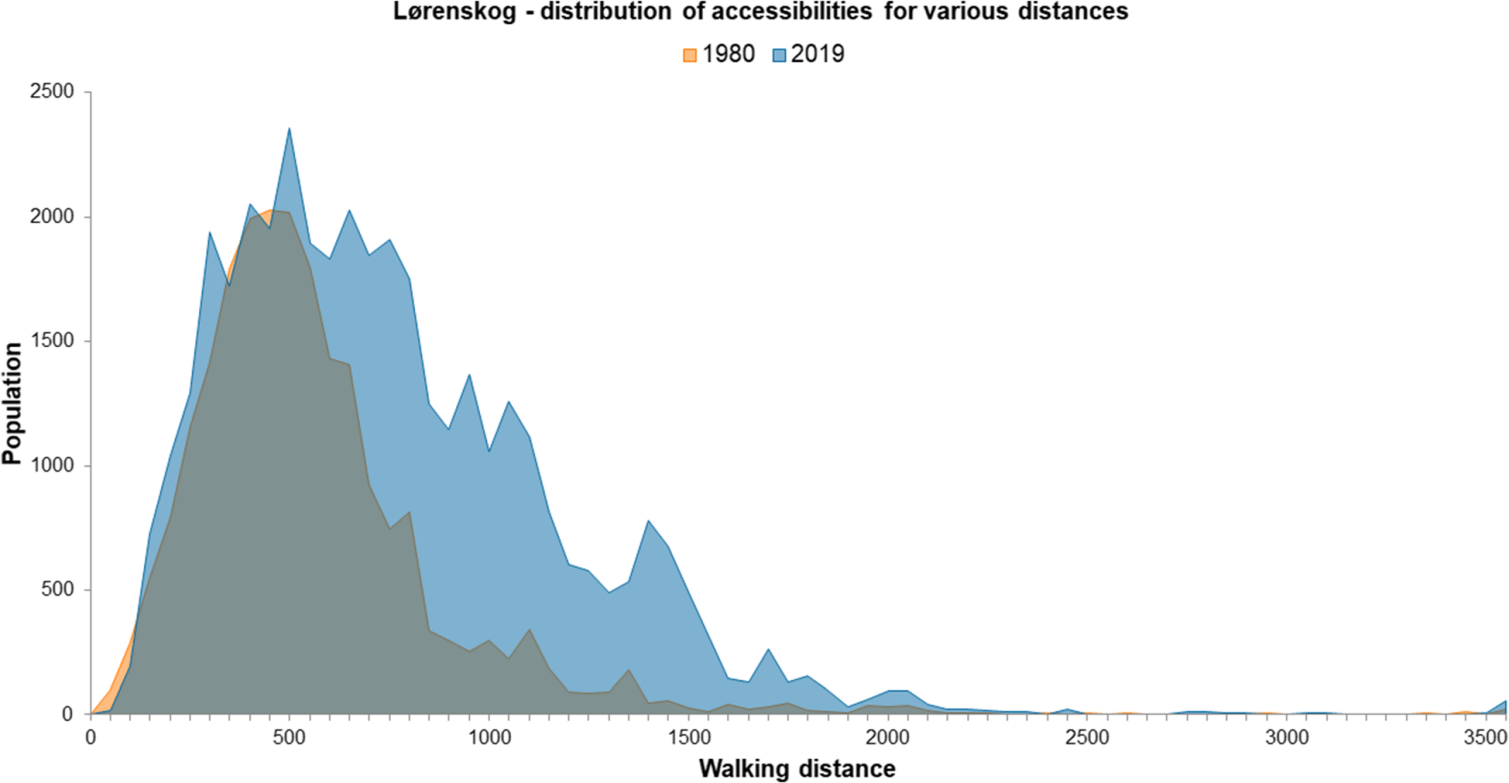
Accessibility from dwellings to grocery stores in Lørenskog, Population at different distances in 50 m intervals, 1980 (orange) and 2019 (blue)

Figures 7 and 8 show the distribution of residents at different walking distances from grocery stores in Hommelvik and Lørenskog, whereas Table 2 shows the same by numbers and percentages of the total population. A clear pattern is the large share of the population in Hommelvik in 1980 having very short distance to grocery stores, 36% living within 500-m walking distance (Table 2), which is the double of the share at current state (19%). The pattern is the same in Lørenskog, the percentage within 500-m walking distance from grocery stores has decreased from 55 in 1980 to 34 in 2019. In Lørenskog, as many as 92% of the population had less than 1 km walking distance to grocery store in 1980, compared to 76% in 2019. We conducted chi-square tests to investigate whether there were significant differences between 1980 and 2019 concerning the number of residents in each of the seven categories. The results were statistically significant for both Hommelvik: χ^2^(6) = 727,678, *p* < 0,001, and Lørenskog: χ^2^(6) = 3518,334, *p* < 0,001. This indicates that there are significant changes in distance between the 2 years at both locations; in both locations the distances are increasing.

The peaks in the diagram (Fig. 7) represent neighbourhoods/clusters of residential buildings at certain distances from grocery stores. In Hommelvik in 1980 there is such clusters at 2000 m distance, representing the then (in 1980) newly developed single-family house area at the east side of the fjord. Due to a grocery store recently built between this area and Hommelvik centre, the accessibility from this area to grocery store has improved. In the curve representing 2019, this area therefore appears as a peak at about 1000 m distance. From the curve representing 2019, we also see two peaks at 2400-3400 m distance. One of these represents the housing areas at top north-east of the map, where many new dwellings are built far outside convenient walking distance to grocery store. The other peak represents the area at the far north-east (top right in the map) where a store existing in 1980 has been closed down.

## Discussion

This paper aimed to identify changes in residential and shopping location and consequent changes in accessibility to the nearest shopping location and found that the proportion of people living within walking distance to a local grocery store has about halved between 1980 and 2019 in the two investigated locations. This represent a fundamental shift in accessibility to a key destination for frequent travel.

Due to the increased distances to nearest shopping location, people have become far more dependent on car for shopping trips; Dependent in the meaning that managing daily life without private car has become more difficult. There could be at least two implications. First, the increased distance is part of the explanation for the strongly increased car use for shopping in Norway (Hjorthol, Engebretsen, & Uteng, 2014). Second, and maybe even more prominent, this change in distances represents a kind of lock-in effect: Increased distances to essential locations make a modal shift from car to more sustainable transport modes difficult to achieve [3, 5, 8]. The changes in accessibility illustrated in this paper are likely parts of structural conditions of locations at neighbourhood-, city- and county scale that are so decisive for travel mode choice that the contrary effects of policies and interventions trying to reduce car driving as currently applied by the government [24, 25], such as increased toll roads for private cars and providing new bicycle lanes, are overruled.

From the perspective of discussing the professional practices of architects and planners, as well as influences from various decisionmakers and stakeholders, it is worth noticing that the kind of accessibility examined in this paper is not an accidental phenomenon but rather the opposite; locations of dwellings and grocery stores as well as positions and layouts of the street network linking these two as pairs of origin-destinations for peoples’ travel, are physical interventions that, with the exception of a few, are intentionally planned, designed and constructed. Therefore, results revealing patterns of change that might be decisive for increased car driving not only provide knowledge about explicit urban form and land use issues that can be improved, such results might also provide useful critique towards methods and practices of planning, design and construction.

Looking in detail at the results, we see two patterns of change causing the increased distance to grocery stores: (1) stores being closed down (often by altering the floor area to dwellings, which is a process requiring acceptance from local planning authorities), and (2) new housing development areas not containing shops. Both cases are closely linked to architecture and planning as professional practices determining layouts and functions of buildings and neighbourhoods, and to the two professions’ contribution in municipal proceedings. A key to improve the conditions for walking and biking is therefore awareness of these patterns of change and active use of this knowledge in their professional practices.

Regarding relevance for the professional practices, also the methods of this study should be of interest. To achieve a future with less car driving and more active mobility, the daily-life accessibility-effects of interventions should be examined more closely than what now is the case. By evaluating alternative development proposals by GIS-based analyses similar to the kind showed here, i.e. mapping explicit accessibility for different modes of transport, and giving priority to projects that clearly improve the conditions for walking and biking at the expense of private cars, a less car-prioritising development should be supported. In cases where analyses reveal that a proposal is unfavourable regarding accessibility by walking and bicycling, there might be many options. In practice, different approaches should likely be combined, for instance ensuring that an area of new development includes a grocery store and that this is located within a context of route network supporting walking and bicycling and ensuring that the number of dwellings within walking distance is sufficient for providing the market of customers needed by the store. All these elements exist in today’s planning practice, but they are scarcely analysed and elaborated together as the strongly interdepended issues of accessibility that they in reality are.

This paper illustrates strengths of GIS based tools both as a means to calculate network distances and for communicating the results. By the kind of modelling of route network applied in this paper, walking (or bicycling) distances are captured more precisely than what would have been the case if relying on open street maps or on the national road network data without correcting these. However, many variables decisive for accessibility on foot are not included, such as slope, traffic safety, standard and maintenance of route and attractiveness and/or recreational value of route [2]. In Hommelvik, the central area along the fjord is flat, with significant hills in all directions from this. This implies that the dwellings outside the central area, for instance in the north-east of the centre (area with buildings coloured blue in the map, Fig. 3) in practice have poorer accessibly to stores than illustrated by distance only. The walking distance to grocery stores in large buildings and particularly in shopping malls, in practice include the walking route from surrounding route network to the building entrance (typically over a car parking area) and also the route inside the building to reach the specific store. Walking distances inside the shopping mall for reaching the grocery store might be long, and exceeding the distance category reported, implying that real distances of 2019 are longer that mapped in our GIS analyses. In addition, our method of mapping does not account for elevations and movement between floors inside a building [6]. The issue of traffic safety is partly handled by roads with heavy traffic and without sidewalks being excluded from the route network model. In reality, the route network model still includes parts where amounts of traffic make walking unsafe or at least uncomfortable, which in turn will likely make people go by car even for short distances. This is probably the case around shopping malls where the entire setting is car prioritising, and in winter for any routes that are not well lit and ploughed free of snow and ice. For more precise assumptions about the effects of the variables currently not or only partly included in the study presented in this paper, larger and more systematic studies are required. Such more nuanced analyses might reveal that the developments in our built environment during recent decades is even more unfortunate than what is found in this paper.

## Conclusion

This paper shows that Norway has experienced a significant increase in walking distances from dwellings to grocery store in the past 40 years. Due to this decreased accessibility to grocery stores by walking or bicycling, the observed increase in car driving for shopping trips should not come as a surprise, and such increased distances to essential locations make a modal shift from car to more sustainable transport modes difficult to achieve. Having the results of this study in mind, together with knowing the current development in Norway regarding intensive construction of new highways, shopping malls and holiday houses, it is likely that the pattern of increased car dependency as found in this study regarding grocery stores may also be present for peoples’ accessibility to other key destinations: We first build highways improving accessibility by car, and then dwellings, shopping malls, and holiday houses exploiting this increased accessibility, which in turn increases car dependency for reaching these locations. Contrary to talks and statements about sustainable urban development and active transportation, it seems that Norway is building a country that in the future will be more rather than less dependent on cars.

## Data Availability

Raw data is not publicly available due to access restrictions set by the mapping authority of Norway. For licenced users, the data can be downloaded from https://kartkatalog.geonorge.no/.

## Declarations

### Journal policy issues

There are no issues relating to journal policies.
